# Further Psychometric Evaluation of the Self-Stigma Scale-Short: Measurement Invariance across Mental Illness and Gender

**DOI:** 10.1371/journal.pone.0117592

**Published:** 2015-02-06

**Authors:** Tsung-Hsien Wu, Chih-Cheng Chang, Chih-Yin Chen, Jung-Der Wang, Chung-Ying Lin

**Affiliations:** 1 Department of Psychiatry, Chi Mei Medical Center, Liouying, Tainan, Taiwan; 2 Department of Psychiatry, Chi Mei Medical Center, Tainan, Taiwan; 3 Health Service and Population Research Department, Institute of Psychiatry, King’s College London, London, United Kingdom; 4 Department of Senior Citizen Service Management, College of Recreation and Health Management, Chia Nan University of Pharmacy and Science, Tainan, Taiwan; 5 Department of Nursing, College of Health Sciences, Chang Jung Christian University, Tainan, Taiwan; 6 Department of Public Health, College of Medicine, National Cheng Kung University, Tainan, Taiwan; 7 Department of Internal Medicine and Occupational Environmental Medicine, National Cheng Kung University Hospital, Tainan, Taiwan; The George Institute for Global Health, INDIA

## Abstract

**Background:**

This study cross-validated the factor structure of the Self-Stigma Scale-Short (SSS-S) in a cohort of patients with mental illness in southern Taiwan. The measurement invariance of the SSS-S factor structure across mental illness and gender was also examined.

**Methods:**

The sample consisted of 161 patients with schizophrenia (51.6% males; mean age ± SD = 40.53 ± 10.38 years) and 189 patients with other mental illnesses (34.9% males; mean age = 46.52 ± 11.29 years).

**Results:**

The internal reliability (total score: α = 0.948) and concurrent validity (*r* = 0.335 to 0.457 with Depression and Somatic Symptoms Scale; *r* = −0.447 to −0.556 with WHOQOL-BREF) of the SSS-S were both satisfactory, and the results verified that the factor structure in our Taiwan sample (RMSEA = 0.0796, CFA = 0.992) was the same as that of the Hong Kong population. In addition, the results supported the measurement invariance of the SSS-S across mental illness (ΔRMSEAs = −0.0082 to −0.0037, ΔCFAs = 0.000) and gender (ΔRMSEAs = −0.0054 to −0.0008, ΔCFAs = −0.001 to 0.000).

**Conclusion:**

Future studies can use the SSS-S to compare self-stigma between genders and between patients with different kinds of mental illnesses.

## Introduction

Self-stigma, aka “internalized stigma” [1], is “one of the especially painful and destructive effects of stigma” [1]. Self-stigma is a transformative process in which a person loses held or desired identities, and adopts a stigmatized and devalued view of himself or herself [2]. In addition, because of the unfriendly environment, e.g., being discriminated against [3] and negatively stereotyped [4], people with a mental illness are prone to develop self-stigma due to social context. Moreover, self-stigma is hypothesized to be negatively related to recovery in people with mental illness [2]. Hence, there is growing interest in the self-stigma of people with mental illness; discussion of this topic has increased over the past few decades [5]. Moreover, many instruments have been developed to measure the self-stigma of people with mental illness [6,7]. Among the self-stigma instruments, the Internalized Stigma of Mental Illness Scale (ISMI) [1] and the Self-Stigma of Mental Illness Scale (SSMIS) [8] are relevant measures with strong reliability and validity [9–11]. In addition, both measures have recently developed a short form for practical use [12,13].

In addition to the ISMI and SSMIS, another instrument, the Self-Stigma Scale-Short (SSS-S) has the following strengths. First, the SSS-S is conceptualized along three psychological dimensions (viz., self-stigmatizing cognition, affect, and behavior) [14], and corresponds to cognitive-behavior theory [15]. Mak and Cheung [14] also examined the construct validity of the SSS-S, and reported that their data fit well with their proposed model. Second, the SSS-S items were generated based on two complementary approaches: focus-group discussion and a comprehensive literature review. The item pool was initially developed by professionals in different fields of psychology (a clinical psychologist, a counseling psychologist, and a social worker), and then its face and content validities and duplication were examined by a team of three graduate students in psychology. Additional psychometric evaluations were done using a group that was different from the group that generated the items. Third, the final version of the SSS-S contains only 9 items, and is feasible for people with mental illness to complete.

However, the validity and reliability of the SSS-S have been examined only in the Hong Kong population, which limits its generalizability to Hong Kong. Because cross-validation in different populations of a newly developed instrument like the SSS-S can strengthen its psychometric properties, we recommend testing the psychometric properties of the SSS-S in different populations (Taiwan, for example). Although Taiwan and Hong Kong share similar traditional cultural values (e.g., Confucianism, Taoism, and Buddhism), they have different social and political systems [16]. Therefore, using the Taiwan population to cross-validate the SSS-S is appropriate. Moreover, Yao and Wu [16] say that there are differences when developing an instrument across subcultures, and that these differences must be considered to provide effective healthcare and treatment.

In addition to the cross-validation of the SSS-S, measurement invariance across mental illness and across gender is important when examining its psychometric properties. Understanding the differences in self-stigma between mental illnesses and genders is useful for healthcare professionals making clinical decisions. However, an important assumption for the comparisons between mental illnesses and genders is measurement invariance [17,18], which means that patients with different mental illnesses do not differently interpret the SSS-S, nor do male and female patients interpret the SSS-S in different ways. To the best of our knowledge, however, only one study [14] has examined the SSS-S, but it did not examine its measurement invariance. The present study, therefore, aimed (1) to establish the internal consistency and concurrent validity of the SSS-S, (2) to cross-validate the theoretical construct of the SSS-S in a Taiwan sample, (3) to test the measurement invariance of the SSS-S across mental illnesses (viz., people with schizophrenia and those with other mental illnesses), and (4) to examine the measurement invariance of the SSS-S across gender.

## Methods

The study was approved by the Research and Ethics Review Board of the Chi Mei Medical Center (IRB: 10102-L06).

### Participants and procedure

Three hundred fifty participants were recruited. All were more than 20 years old, were outpatients with mental disorders, inpatients of psychiatric acute wards, patients of psychiatric daycare, or patients with mental disorders receiving homecare services from Chi Mei Medical Center. All could read, speak, and understand spoken Mandarin Chinese, and all volunteered to participate. The participants were approached in the following manner: First, the psychiatrists identified patients who met the inclusion criteria. Second, the psychiatrists briefly introduced and explained the study to these potential participants, and referred those who were interested in the study to several research assistants. Third, the research assistants gave a detailed introduction of the study to the participants, and then requested signed informed consents from those who were willing to participate. Finally, 350 participants signed written informed consents. All were evaluated by one psychiatrist and a psychiatric medical group to confirm that they were competent to consent and to participate in this study. Furthermore, none of the participants had been diagnosed with dementia, mental retardation, an organic mental disorder, or autism. We wanted the participants to freely sign the informed consents by themselves; none of them used a surrogate consent. The participants then, under the supervision of several research assistants, completed the Self-Stigma Scale-Short (SSS-S), the Depression and Somatic Symptoms Scale (DSSS), the WHO questionnaire on the Quality of Life, the Short Form (WHOQOL-BREF), and one background information sheet. Both the DSSS and the WHOQOL-BREF were used to test the concurrent validity of the SSS-S. Because patients with mental illness are easily depressed and generally have a worse quality of life (QoL) due to their stigma [2,19–21], we hypothesized that the SSS-S would be moderately correlated with the DSSS and with the WHOQOL-BREF. In addition, the diagnoses of all participants were collected from their medical records.

### Instrumentation

**Self-Stigma Scale-Short (SSS-S)**

The self-rated SSS-S contains only 9 items about self-stigma in three dimensions (viz., cognition, affect, and behavior) each with 3 items. The SSS-S is designed for different minority groups (e.g., mental health consumers, immigrants, and sexual-orientation minorities: lesbians, gay men, and bisexuals), and the terms describing the minority group being tested can be replaced. Because all the participants of this study had a mental illness, we used the term “mental illness” in the SSS-S. Each item asks the respondents to rate their agreement on a 4-point Likert scale from 1 (strongly disagree) to 4 (strongly agree). In addition, the reliability (α = 0.87), the concurrent validity, and the construct validity (comparative fit index; CFI = 0.97) have been supported for patients with mental illness [14].

**Depression and Somatic Symptoms Scale (DSSS)**

The DSSS is a self-rated questionnaire, and has a Depression domain with 12 items, and a Somatic domain with 10 items. The items are rated using a 4-point Likert Scale, and ask how serious the symptom is (0: not at all, 1: mild, 2: moderate, 3: severe). Therefore, a higher score in the DSSS represents a worse condition. In addition, the concurrent validity and the reliability (α = 0.73 to 0.94, *r* = 0.88 to 0.92 for test-retest) of the DSSS Chinese version have been examined [22].

**The WHO questionnaire on the Quality of Life, Short Form (WHOQOL-BREF)**

The WHOQOL-BREF Taiwan version has 28 items with four domains (Physical: 7 items, Psychological: 6 items, Social: 4 items, and Environmental: 9 items). All items have 5-point self-rated scales with a higher score representing a better QoL. The validity (CFI = 0.89) and the reliability (03B1 = 0.70 to 0.91, *r* = 0.76 to 0.80 for test-retest) of the WHOQOL-BREF Chinese version have been tested [23].

### Statistical Analysis

The descriptive analyses, the internal consistency (i.e., Cronbach’s α), and the concurrent validity using Pearson correlation coefficients were analyzed using SPSS 16.0 for Windows (SPSS Inc., Chicago, IL, USA). The confirmatory factor analyses (CFAs), including measurement invariance, were done using LISREL 8.8 for Windows (SSI Inc., Lincolnwood, IL, USA).

Because all the items in the SSS-S were normally distributed (skewness = −0.111 to 0.802; kurtosis = −1.008 to 0.376), a maximum likelihood estimation was used for all CFAs. A second-order model was used for the whole sample and for the separate samples (viz., the sample with schizophrenia, the sample with other mental illnesses, the male sample, and the female sample). The second-order model was also used to evaluate measurement invariance, and the 10 models were as follows:

Model 1M/1G: configural model for mental illnesses/genders;
Model 2M/2G: all first-order factor loadings were invariant between mental illnesses/genders;
Model 3M/3G: all first-order factor loadings and item intercepts were invariant between mental illnesses/genders;
Model 4M/4G: all first- and second-order factor loadings and item intercepts were invariant between mental illnesses/genders;
Model 5M/5G: all first- and second-order factor loadings, item intercepts, and construct means were invariant between mental illnesses/genders;

Fit indices of a nonsignificant χ^2^ statistic, root mean square error of approximation (RMSEA) < 0.08, comparative fit index (CFI) > 0.95, and standardized root mean square residual (SRMR) < 0.08 were used to determine whether the data-fit of the model was satisfactory [24,25]. Moreover, goodness of fit (GFI), adjusted goodness of fit (AGFI), Akaike’s information criteria (AIC), and consistent Akaike’s information criteria (CAIC) were also reported for the second-order models of four separate samples. A nonsignificant χ^2^ statistic was also used to test measurement invariance. In addition, ΔRMSEA and ΔCFI < 0.01 suggest that factor loadings, item intercepts, and construct means were invariant across measurements. ΔSRMRs < 0.03 and < 0.01 also suggest that factor loadings and item intercepts were invariant [17,26–29].

## Results

The mean age (± standard deviation) was 40.53 ± 10.38 years for the participants with schizophrenia, and 46.52 ± 11.29 years for those with other mental illnesses. The age at onset was 26.69 ± 8.85 years for the participants with schizophrenia, and 36.31 ± 12.25 years for those with other mental illnesses. About half of the participants with schizophrenia were female (78/161 = 48.4%), and nearly two-thirds with other mental illnesses were female (123/189 = 65.1%). More than half of the participants with schizophrenia were single (112/161 = 69.6%), and more than half with other mental illnesses were currently married (110/189 = 58.2%). Of the participants of other mental illnesses, 52.4% (99/189) were diagnosed with depressive disorder, 23.3% (44/189) with bipolar disorder, 18.0% (34/189) with anxiety disorder, and the remaining 6.3% (12/189) with still other mental illnesses (Table 1).

**Table 1:** Demographic data.

Characteristics	Schizophrenia (*n* = 161)		Other mental illnesses (*n* = 189)	
	Mean or (*n*)	SD or %	Mean or (*n*)	SD or %
Age (years)	40.53	10.38	46.52	11.29
Age at onset (years)	26.69	8.85	36.31	12.25
Gender				
Male	(83)	51.6%	(66)	34.9%
Female	(78)	48.4%	(123)	65.1%
Education				
Junior high or less	(34)	21.1%	(67)	35.4%
Senior high	(78)	48.4%	(69)	36.5%
College or higher	(49)	30.4%	(53)	28.0%
Marital status				
Currently married	(29)	18.0%	(110)	58.2%
Single	(112)	69.6%	(45)	23.8%
Other	(20)	12.4%	(34)	18.0%
Diagnoses				
Schizophrenia & other psychosis	(161)	100.0%	—	—
Depressive disorder	—	—	(99)	52.4%
Bipolar disorder	—	—	(44)	23.3%
Anxiety disorder	—	—	(34)	18.0%
Others	—	—	(12)	6.3%

SD, standard deviation

The internal consistency was good in three domain scores of the SSS-S (α = 0.878 in Cognition, 0.802 in Affect, and 0.913 in Behavior). The total score of the SSS-S also had a high internal consistency of α = 0.948. In addition, the SSS-S scores were positively and moderately correlated with the DSSS scores (*r* = 0.335 to 0.457, *P* < 0.01), and negatively and moderately correlated with the WHOQOL-BREF scores (*r* = −0.447 to −0.556, *P* < 0.01) (Table 2).

**Table 2:** Pearson correlation coefficients between the SSS-S, the DSSS, and the WHOQOL-BREF.

	SSS-S		
	Cognitions	Affect	Behaviors
DSSS			
Depression	0.424	0.405	0.457
Somatic	0.355	0.335	0.370
WHOQOL-BREF			
Physical	−0.522	−0.464	−0.479
Psychological	−0.556	−0.550	−0.550
Social	−0.450	−0.447	−0.477
Environmental	−0.465	−0.454	−0.478

All *P*-values < 0.01

SSS-S = Self-Stigma Scale-Short

DSSS = Depression and Somatic Symptoms Scale

WHOQOL-BREF = The WHO questionnaire on the Quality of Life, Short Form

Except for the significant χ^2^ statistic (χ^2^ = 68.278, *df* = 22, *P* < 0.01), the data-model fit indices were all satisfactory for the whole sample in our proposed second-order CFA model (CFI = 0.992, RMSEA = 0.0796, and SRMR = 0.0249). For separate baseline models, all standardized factor loadings and construct means were significant for the participants with schizophrenia and other mental illnesses, and for male and for female participants (Table 3). In addition, except for all significant χ^2^ statistics (χ^2^ = 46.150 to 63.861, *df* = 22, *P* < 0.01) and some RMSEAs (0.0814 to 0.1120), all fit indices were excellent for the participants with schizophrenia, the participants with other mental illnesses, the male participants, and the female participants (Table 3).

**Table 3:** Standardized factor loadings, construct means, and model fit for participants stratified by gender and type of mental illness.

	Mental illness		Gender	
	Schizophrenia	Others	Male	Female
Factor loadings				
Self-stigma				
Cognition	0.93	0.96	0.92	0.95
Affect	1.00	1.00	1.00	1.00
Behavior	0.98	0.95	0.95	0.97
Cognition				
C1	0.86	0.87	0.89	0.86
C2	0.84	0.86	0.79	0.89
C3	0.84	0.86	0.85	0.86
Affect				
A1	0.75	0.80	0.74	0.82
A2	0.86	0.89	0.86	0.90
A3	0.66	0.64	0.67	0.63
Behavior				
B1	0.87	0.91	0.89	0.90
B2	0.86	0.84	0.80	0.89
B3	0.80	0.84	0.75	0.87
Construct means				
Cognition	2.38	2.26	2.30	2.36
Affect	2.07	1.93	1.93	2.05
Behavior	2.24	2.12	2.05	2.09
Fit indices				
χ^2^ (*df*)	46.150 (22)	49.121 (22)	63.861 (22)	48.602 (22)
GFI/AGFI	0.940/0.878	0.945/0.887	0.913/0.823	0.949/0.896
AIC/CAIC	91.61/185.48	95.43/192.99	109.15/201.24	94.29/193.27
RMSEA/CFI	0.0819/0.990	0.0814/0.0991	0.1120/0.981	0.0773/0.992
SRMR	0.0306	0.0282	0.0417	0.0222

All *P-*values of factor loadings, construct means, and χ^2^ of four models were < 0.01; *df* = degree of freedom; RMSEA = root mean square error of approximation; CFI = comparative fit index; SRMR = standardized root mean square residual; GFI = goodness of fit index; AGFI = adjusted goodness of fit index; AIC = Akaike’s information criterion; CAIC = consistent Akaike’s information criterion

The fit indices for measurement invariance were all acceptable across the participants with schizophrenia and those with other mental illnesses (Δχ^2^ = 0.376 to 3.820, *P* = 0.70 to 0.95, ΔRMSEA = −0.0082 to −0.0037, ΔCFI = 0.000, and ΔSRMR = 0.0004 to 0.0028), except for one significant Δχ^2^ statistic (Δχ^2^ = 10.982, *P* = 0.01) that examined the invariance of construct means (Tables 4 and 5). In addition, all data-model fit indices except the significant χ^2^ statistic for Model 5M vs. Model 4M were satisfactory (Table 5).

**Table 4:** Measurement invariance across schizophrenia and other mental illnesses, and across genders.

	Fit indices					
Model	χ^2^	df	*P*-value	RMSEA	CFI	SRMR
Mental illness						
1M	95.270	44	< 0.01	0.0817	0.991	0.0282
2M	97.693	50	< 0.01	0.0735	0.991	0.0310
3M	101.513	56	< 0.01	0.0674	0.992	0.0314
4M	101.889	59	< 0.01	0.0637	0.992	0.0336
5M	112.871	62	< 0.01	0.0679	0.991	0.0318
Gender						
1G	112.463	44	< 0.01	0.0939	0.988	0.0222
2G	121.201	50	< 0.01	0.0885	0.987	0.0344
3G	130.139	56	< 0.01	0.0849	0.987	0.0330
4G	132.598	59	< 0.01	0.0823	0.987	0.0651
5G	137.578	62	< 0.01	0.0815	0.987	0.0653

Model 1M/1G: Configural model

Model 2M/2G: All first-order factor loadings were invariant between mental illnesses (2M) and genders (2G)

Model 3M/3G: All first-order factor loadings and item intercepts were invariant between mental illnesses (3M) and genders (3G)

Model 4M/4G: All first- and second-order factor loadings, and item intercepts were invariant between mental illnesses (4M) and genders (4G)

Model 5M/5G: All first- and second-order factor loadings, item intercepts, and construct means were invariant between mental illnesses (5M) and genders (5G)

*df* = degree of freedom; RMSEA = root mean square error of approximation; CFI = comparative fit index; SRMR = standardized root mean square residual; GFI = goodness of fit index; AGFI = adjusted goodness of fit index; AIC = Akaike’s information criterion; CAIC = consistent Akaike’s information criterion

**Table 5:** Model comparisons across schizophrenia and other mental illnesses, and across genders.

Models compared	Δχ^2^	Δ*df*	*P*-value	ΔRMSEA	ΔCFI	ΔSRMR
Mental illness						
2M vs. 1M	2.423	6	0.88	−0.0082	0.000	0.0028
3M vs. 2M	3.820	6	0.70	−0.0061	0.001	0.0004
4M vs. 3M	0.376	3	0.95	−0.0037	0.000	0.0022
5M vs. 4M	10.982	3	0.01	0.0042	−0.001	−0.0018
Gender						
2G vs. 1G	8.738	6	0.19	−0.0054	−0.001	0.0122
3G vs. 2G	8.938	6	0.18	−0.0036	0.000	−0.0014
4G vs. 3G	2.459	3	0.48	−0.0026	0.000	0.0321
5G vs. 4G	4.980	3	0.17	−0.0008	0.000	0.0002

Model 1M/1G: Configural model

Model 2M/2G: All first-order factor loadings were invariant between mental illnesses (2M) and genders (2G)

Model 3M/3G: All first-order factor loadings and item intercepts were invariant between mental illnesses (3M) and genders (3G)

Model 4M/4G: All first- and second-order factor loadings, and item intercepts were invariant between mental illnesses (4M) and genders (4G)

Model 5M/5G: All first- and second-order factor loadings, item intercepts, and construct means were invariant between mental illnesses (5M) and genders (5G)

*df* = degree of freedom; RMSEA = root mean square error of approximation; CFI = comparative fit index; SRMR = standardized root mean square residual

The fit indices for measurement invariance were all acceptable across the male and female participants (Δχ^2^ = 2.459 to 8.938, *P* = 0.17 to 0.48, ΔRMSEA = −0.0054 to −0.0008, ΔCFI = −0.001 to 0.000, and ΔSRMR = −0.0014 to 0.0122), except for one slightly high ΔSRMR value of 0.0321 that examined the invariance of second-order factor loadings (Table 5). In addition, all data-model fit indices except the significant χ^2^ statistic and slightly high values of RMSEA (0.0815 to 0.0939) were satisfactory (Table 4).

## Discussion

This is, to the best of our knowledge, the first study to examine any self-stigma instrument for measurement invariance across mental illnesses. Although many studies have evaluated several self-stigma instruments and verified their psychometric properties [6,7], none of them examined the measurement invariance across mental illnesses. Therefore, except for the SSS-S, which we found to be measurement invariant across mental illnesses, other self-stigma instruments have not been verified to compare groups with different mental illnesses. Other psychometric properties of the SSS-S were also supported in our Taiwan sample.

We found satisfactory internal consistency (α = 0.802 to 0.948) for the SSS-S, and a moderate and significant correlation between the SSS-S, the DSSS (*r* = 0.335 to 0.457), and the WHOQOL-BREF (*r* = −0.447 to −0.556). Therefore, the validity and reliability of the SSS-S Taiwan version have been confirmed. In addition, the satisfactory data-model fit indices support the second-order structure of the SSS-S construct, and indicate that the SSS-S has good construct validity. All of these psychometric results are consistent with Mak and Cheung [14], who found that the α of the SSS-S was 0.807 and that the SSS-S score was moderately correlated with perceived stigma (*r* = 0.54, *P* < 0.01). They also found a satisfactory data-model fit for the second-order model of the SSS-S (CFI = 0.97, RMSEA = 0.06), and their data-model fit results agree with our findings. In addition, the significant factor loadings reported by Mak and Cheung are comparable to ours. Standardized factor loadings (Mak and Cheung vs. ours) of Self-stigma on Cognition were 0.88 vs. 0.92–0.95, of Self-stigma on Affect were 1.00 vs. 1.00, of Self-stigma on Behavior were 0.93 vs. 0.95–0.98, of Cognition on items were 0.62–0.82 vs. 0.79–0.89, of Affect on items were 0.62–0.83 vs. 0.63–0.90, of Behavior on items were 0.54–0.78 vs. 0.75–0.91.

We found that the SSS-S was measurement invariant across mental illnesses, except for the significant Δχ^2^ value that tested for construct means (i.e., means of Cognition, Affect, and Behavior domains). However, we claim that the construct means are invariant across mental illnesses for the following reasons. First, χ^2^ difference tests have been verified to have the shortcoming of being too sensitive to a large sample size [17,24,26]. With a sample size > 300 in our study, it is easy for the χ^2^ value to be significant. Second, all other alternative values (i.e., ΔRMSEA, ΔCFI, and ΔSRMR) fulfilled the invariance criteria. Because measurement invariance is the assumption for comparing or combining different groups [17,18], when it is supported for an instrument, healthcare professionals can use that instrument to compare the self-stigma between mental illnesses. The results of supporting measurement invariance for the SSS-S will benefit future studies comparing or combining samples with different mental illnesses. Because of the growing interest in the self-stigma of people with mental illness [5], related discussions are presented on specific mental illness [30,31], and combined using different kinds of mental illnesses [32]. Moreover, healthcare professionals may be interested in comparing self-stigma between people with different mental illnesses. Therefore, we conclude that, because its measurement invariance across mental illness is supported, the SSS-S is a viable instrument for comparing self-stigma between people with different mental illnesses.

Some studies have explored and compared self-stigma between genders [33,34]. We found that the SSS-S was measurement invariant across gender. Although one index (i.e., ΔSRMR = 0.0321) used for testing the invariance of second-order factor loadings was slightly higher than the criterion of 0.025, we believe that the second-order factor loadings still can be seen as invariant across genders. One reason is that the ΔSRMR value was very close to the criterion (the difference between the values of our ΔSRMR and the criterion was 0.0071), while the other indices (both the ΔRMSEA and ΔCFI < 0.01) were satisfactory. Another reason is that Model 4G had an acceptable SRMR value of 0.0651; thus, Model 4G was supported as having second-order factor loadings invariant across genders. Therefore, health professionals can use the SSS-S to compare self-stigma between male and female patients with mental illness.

However, healthcare professionals should still interpret our measurement invariance with caution because the results of two of our criteria slightly violated the recommendation. Specifically, people with schizophrenia tended to have higher construct means in self-stigma (Cognition: 2.38, Affect: 2.07, and Behavior: 2.24) than those with other mental illnesses (Cognition: 2.26, Affect: 1.93, and Behavior: 2.12), which could be an indication that people with schizophrenia encounter more social stigma than those with other mental illnesses. In addition, female participants seemed to show higher factor loadings of cognition and behavior in self-stigma than did males, which may indicate that female participants considered their illnesses a more serious problem and withdrew more easily than did their male counterparts. Therefore, healthcare providers might want to more deeply investigate the perceived stigma in people with schizophrenia, and the thoughts of females with mental illnesses.

This study has some limitations. First, we had only two mental illness groups: the schizophrenia group and the other mental illness group. This is an inexact and unbalanced bifurcation because all the members of one group had been diagnosed with the same mental illness, but the members of the other group consisted of people with miscellaneous mental illnesses. Although we recommend that the best way to test the measurement invariance across mental illnesses is to create separate groups of participants, each with a specific mental illness, in this study, we decided against this strategy because the sample sizes for each group were too small (*n* = 99 for depressive disorder, 44 for bipolar disorder, and 34 for anxiety disorder). Several studies [35–38] suggest an *n* of at least 100 for CFA, which persuaded us to separate our participants into two groups to fulfill the sample-size requirement. Although our results show a good data-model fit, which somewhat supports our decision as appropriate, future studies need to test the measurement invariance across separate specific mental illnesses. To shed some light on future research, we did a further measurement invariance test across people with schizophrenia (*n* = 161) and people with mood disorders (depression and bipolar disorder; *n* = 143). The results of the additional examination support the measurement invariance (Δχ^2^ = 0.353 to 5.274, *P* = 0.15 to 0.95, ΔRMSEA = −0.0082 to 0.0013, ΔCFI = −0.002 to 0.002, and ΔSRMR = −0.0028 to 0.0064). Second, the representativeness of our results may be limited to part of the Taiwan population because all participants were from southern Taiwan.

Third, because Hong Kong and Taiwan use the same orthographic system, we did not use the standard procedure of forward translation, reconciliation, and backward translation to translate the SSS-S; therefore, the linguistic validity may be jeopardized [39]. Although the major Chinese dialects spoken in Hong Kong (Cantonese) and Taiwan (Mandarin and Taiwanese) are pronounced differently and are mutually incomprehensible when spoken, Hong Kong’s Cantonese and Taiwan’s Mandarin use almost the same set of written traditional Chinese characters, not the simplified character set used by the People’s Republic of China. Although some terms may have slightly different meanings between Hong Kong and Taiwan, our second author has discussed the issue with Dr. Mak, the developer of the SSS-S, and confirmed that the orthographic systems between Hong Kong and Taiwan are consistent. In addition, our psychometric results show that the internal consistency, concurrent validity, and construct validity of the SSS-S Taiwan version are satisfactory; thus, we are confident that the linguistic validity problem was not serious. Fourth, the DSSS and WHOQOL-BREF, the scales we used for testing the concurrent validity, might not be “gold standards” for measuring self-stigma, because there are at least two other instruments with strong reliability and validity: the ISMI and the SSMIS. However, because other studies [2,19–21] found that depression (which can be measured by DSSS) and QoL (which can be measured by WHOQOL-BREF) are correlated with self-stigma, we simply compared the concurrent validity with these two scales, and they showed moderate correlations in our study. Future studies to explore the correlation between the SSS-S and the ISMI or SSMIS, or both, are required for a more comprehensive validation.

In sum, the Taiwan version of the SSS-S is a valid and reliable instrument for clinical healthcare professionals to use for measuring and evaluating self-stigma for people with mental illness. They can use the SSS-S to compare self-stigma between people with different mental illnesses and different genders. Moreover, the SSS-S can also be used to examine the effect of programs on decreasing self-stigma for people with mental illness. Although we provided vigorous psychometric evidence for the SSS-S, its psychometric properties were examined only in Asia. Therefore, additional psychometric studies on the SSS-S in other cultures are recommended. Future studies may also want to stratify mental illnesses and to ensure a proper number of participants (say, 100 or more) per disorder. Rigorous results of measurement invariance can then be examined.
